# High-yield grass *Pennisetum sinese Roxb* plantation and organic manure alter bacterial and fungal communities structure in an ecological agriculture farm

**DOI:** 10.1186/s13568-020-01018-2

**Published:** 2020-05-06

**Authors:** Yan He, Lu Lu, Chao Peng, Huilin Li, Jing Zhang, Run Li, Caiquan Zhou

**Affiliations:** 1 Key Laboratory of Southwest China Wildlife Resources Conservation, College of Life Sciences, China West Normal University, Nanchong, 637002 China; 2 Institute of Ecology, China West Normal University, Nanchong, 637009 China; 3 College of Environmental Science and Engineering, China West Normal University, Nanchong, 637009 China

**Keywords:** *Pennisetum sinese Roxb*, Organic manure, Illumina Miseq sequencing, Bacterial and fungal differentiation

## Abstract

*Pennisetum sinese Roxb* (*P. sinese*) is an efficient and economic energy crop for its high productivity, and has been well studied in its application in phytoremediation and fodder production. However, little is known about how *P. sinese* plantation and fermented manures of *P. sinese*-feed livestock affect the composition of soil bacterial and fungal communities. In this study, 16S rRNA/ITS1 gene-based Illumina Miseq sequencing was employed to compare the bacterial and fungal community structure among soils that had been subjected to uncultivated control (CK), 2-year *P. sinese* plantation (P), and *P. sinese* plantation combined with the use of organic manures (P-OM) in a “*P. sinese*—breeding industry” ecological agriculture farm. The results found microbial communities were altered by *P. sinese* plantation and fertilization. The *P. sinese* plantation resulted in increased *Actinobacteria* and *Planctomycetes* abundance. Comparatively, significant increased abundance of *Chloroflexi*, *Firmicutes*, *Nitrospirae*, and *Euryarchaeota*, and genes related with nitrogen and carbon metabolic pathways based on PICRUSt prediction was observed in P-OM soil. Fungal compositions suggested a markedly increased abundance of *Ascomycota* in P soil. Potential organic matter decomposers *Candida*, *Thermoascus*, and *Aspergillus* were enriched in *P* soil, indicating the enhanced role of fungi in litter decomposition. Redundancy analysis suggested that soil properties (NH_4_^+^-N, total nitrogen, organic matter content, and soil water content) significantly correlated with the changes of microbial compositions (*P* < 0.05). These results highlight the divergence of microbial communities occurs during *P. sinese*-based plantation, implying functional diversification of soil ecosystem in *P. sinese* fields.

## Introduction

**Fig. 1:**
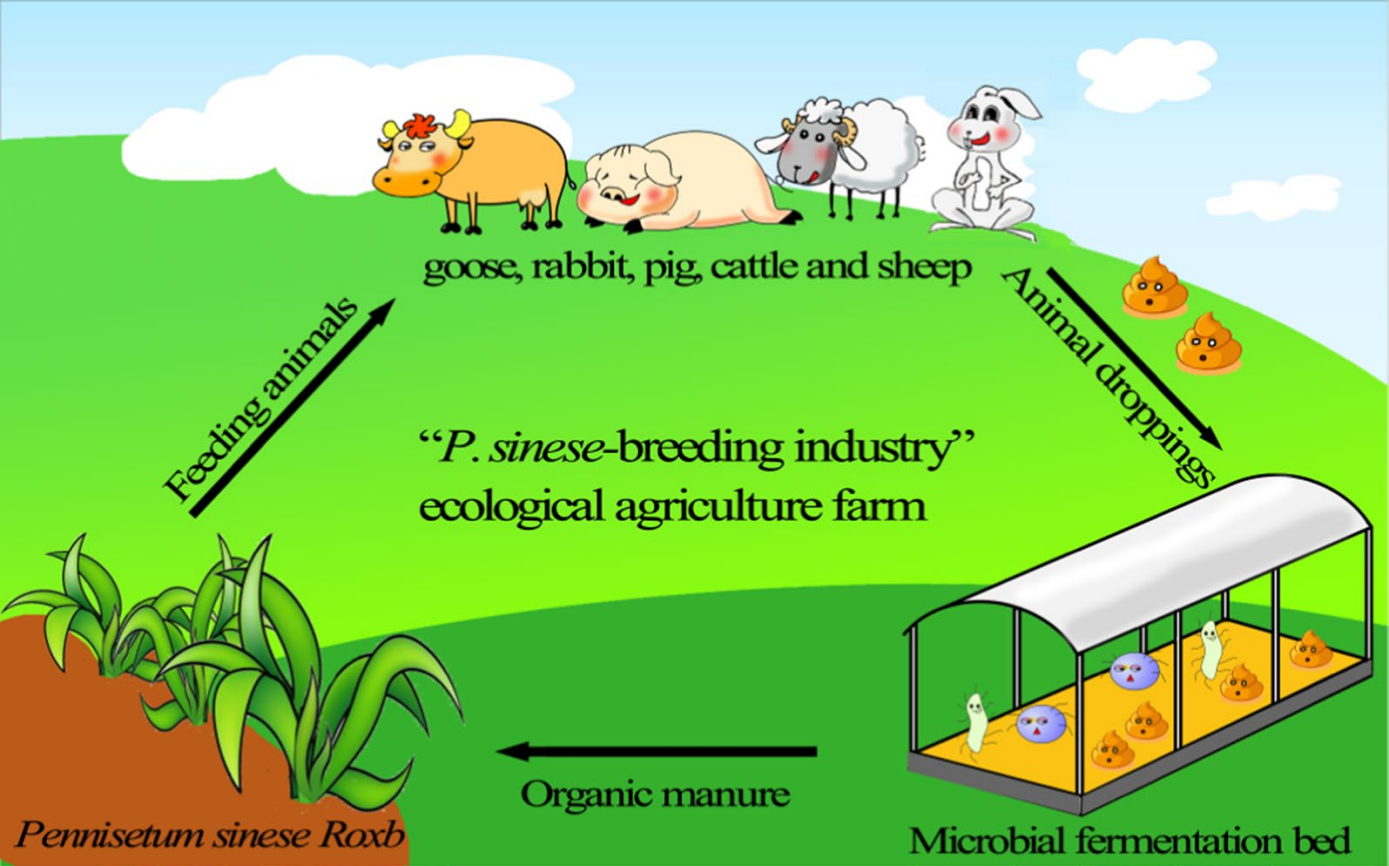
Cartoon diagram of the“*Pennisetum sinese Roxb*—breeding industry” “*P. sinses*—breeding industry” ecological agriculture farm. The diagram was designed and drawn by Yan He

The development of ecological agriculture has been regarded as an important mean to realize the coordinated development of the environment and economy, which is particularly the case in China (Shi and Gill 2005). Among the ecological agriculture development models, the model of “*P. sinese*—breeding industry” is one of common practices which has been established in many farms and villages (Chen et al. 2012; Wei 2005). *P. sinese*, widely known as giant bamboo grass or king grass, is a type of fast-growing perennial and monocot C_4_ plant with highly developed root systems, high yield of stems and leaves, and high level of protein and sugar contents (Lu et al. 2014). *P. sinese* with strong adaptability is widely planted and extensively distributed in tropical and subtropical regions. These capacities have carved a niche for itself in phytoremediation (Chen et al. 2017), soil reclamation (Senlin et al. 2017), and fodder source (Yongfen 1999). In these farms, fresh stem leaves of *P. sinese* are fed to livestock, and then the fermented livestock manures as organic matter are applied into arable fields to maximize the crop yields (He et al. 2012) (as illustrated in Fig. 1 and Additional file 1: Fig. S1). Previous studies revealed that *P. sinese* plantation could be beneficial for enhancing the soil fertility (Xu et al. 2015; Lin et al. 2014). Fundamentally, it is microorganisms inhabiting soil ecosystem play crucial roles in maintaining soil ecological functions, including maintaining ecosystem health and nutrients biogeochemical cycling (Rick and Thomas 2001). However, little is known about how *P. sinese* plantation and organic manure of *P. sinese*-feed livestock affect the composition of microbial communities and their roles in maintaining soil productivity.

Microbial composition and diversity are manipulated by environment conditions (Singh et al. 2009), which is reflected in the high sensitivity of microorganisms to anthropogenic or natural disturbance (Yin et al. 2010). A number of studies have shown that shifts of bacterial and fungal community structure are related to the alteration of soil nutrient contents (van der Wal et al. 2006; Gravuer and Eskelinen 2017) and soil pH (Shen et al. 2013). Vegetation type is another main factor in shaping microbial communities (Nielsen et al. 2010). This is quite reasonable that vegetation could alter soil physicochemical and biotic parameters by secreting root exudate (such as amino acids, sugars, polysaccharides or proteins) (Badri and Vivanco 2009), accumulating root residues (Shahbaz et al. 2017), and preferential assimilation of nutrient substrates (Bai et al. 2017), and thus act as a selective force to drive the diversification of microbial communities. It has been reported that *P. sinese* can stimulate the enzyme activities of soil microbial populations (Cui et al. 2016), and enhance microbial community functional diversity (Lin et al. 2014). Moreover, *P. sinese* showed a great uptake capacity of nitrogen and phosphorus from wetland (Xu et al. 2015). However, it remains to be seen whether the soil bacterial and fungal communities response to plantation of *P. sinese*.

Increasing lines of evidences have suggested that livestock manures as organic fertilizers could exert a remarkable influence on altering the microbial communities (Zhong et al. 2010; Dumontet et al. 2017). For example, addition of livestock manures increased microbial diversity (Sun et al. 2015), which could even be detected within a short period of fertilization (Lazcano et al. 2013). Hence, an attempt to determine the shifts in bacterial and fungal patterns caused by the fermented manures from *P. sinese*-feed livestock have also been explored in the present study.

We hypothesized that the soil microbial communities response strongly to the plantation of *P. sinese* and application of livestock manures, and show a significant correlation of soil microbial community compositions with soil physicochemical parameters. Here, soils with a *P. sinese* cultivation and fertilization history of 2 year in an ecological farm were selected to investigate the community diversification of bacteria and fungi using 16S rRNA/ITS1 gene-based Illumina MiSeq sequencing.

## Materials and methods

### Site description and soil sampling

The soils were collected from the experimental field at Huangjialou Village ecological farm where a recycling system was established in 2016 (30° 29′ N, 106° 40′ E). The village is located in Nanchong City of Sichuan Province in southwest China. Huangjialou village is also one of the key villages which are listed in poverty alleviation project in China. This region has a typical mid-subtropical humid monsoon climate with a mean annual temperature of 17.0 °C. The soil sampling sites were converted from abandoned land to *P. sinese* planting field for 2 years. The harvested *P. sinese* is used for feeding animals (chicken, goose, rabbit, cattle, and sheep). The manures from animals are fermented, and then spread as organic fertilizers for *P. sinese* and other crops in each season, at ~ 200.0 kg ha^−1^ (fresh weight). Three sample plots received different treatments consisting of (1) an uncultivated abandoned land control (CK); (2) 2-year *P. sinese* planting (P); (3) a combination of 2-year *P. sinese* planting and organic manure fertilization (P-OM). Each plot sampling was conducted in triplicate.

Soil sampling was performed using a hand trowel at a depth of 0–10 cm. Plant residues and other materials, such as visible macrofauna and stones, were removed before the sampling. The soils were placed in sterile plastic bags, sealed, and transported to the laboratory within 2 h. Soils were stored at − 20 °C for physicochemical and molecular analysis.

### Sample physicochemical analysis

The soil pH was measured by a pH meter (Mettler-Toledo Instruments Co. Ltd., Shanghai, China) using a soil-to-water ratio of 1:2.5. The soil organic matter (SOM) content was determined using a total carbon analyzer (TOC-V CPH, Shimadzu, Japan). Total nitrogen (TN) was determined using the Kjeldahl digestion method (Bremner 1965). The soil water content (SWC) was measured by drying the fresh sample at 105 °C for 6 h. 5 g of fresh soil was homogenized with 20 ml of 2 M KCl by shaking at 150 r.p.m for 60 min, and then passed through a Whatman^®^ Grade 1 qualitative filter paper (circles, diam. 110 mm) for the determination of NH_4_^+^-N, NO_2_^−^-N, and NO_3_^−^-N using a Skalar SAN Plus segmented flow analyser (Skalar, Breda, the Netherlands). All the soil parameters were calculated based on oven-dried soil weight. All samples were analyzed in three replicates.

### Soil DNA extraction and real-time quantitative PCR

Total DNA extraction from the soils was performed using a FastDNA spin kit for soil (Qbiogene, Irvine, CA). 0.5 g soil was used directly for DNA extraction. The quality and quantity of extracted DNA were determined using a NanoDrop spectrophotometer (NanoDrop Technologies, Wilmington, Germany). The DNA was stored at − 20 °C until use.

### Illumina Miseq sequencing of 16S rRNA and ITS1 gene amplicons

Illumina Miseq sequencing was employed to investigate the shifts of bacterial and fungi communities in CK, P, and P-OM soils. The primer pairs 515/907 and ITS 5F/1R were used for the amplification of bacterial 16S rRNA and fungal ITS1 genes, respectively. The PCR primers and conditions are listed Additional file 1: Table S1. All PCR amplifications were conducted in triplicate for each treatment, and then visualized on 1.0% agarose gels with GoldView™. The concentration of the purified PCR amplicons was determined, and these amplicons were used for the Miseq sequencing. The Miseq sequencing data were analysed using the QIIME 2 software package (Bokulich et al. 2017). The detailed analysis for sequencing was performed as described previously (Wang et al. 2018).

### Statistical analysis

The chemical properties data of the soils were compared though multiple sample comparisons using one-way ANOVA analysis to check for quantitative variances among different soil samples. SPSS version 20.0 was used for statistical analysis (SPSS Inc., Chicago, IL, USA). Correlations between soil physicochemical properties and bacterial, fungal community compositions were carried out using CANOCO 4.5 software package (Biometris, Wageningen, The Netherlands). Bacterial and fungal community comparisons at phylum and genus level were performed using Metastats (White et al. 2009). Bacterial functional gene prediction was determined using Phylogenetic Investigation of Communities by Reconstruction of Unobserved States (PICRUSt) (Langille et al. 2013).

## Result

### Soil properties

The *P. sinese* plantation and organic manures did not result in a significant change in pH values from 8.43 ± 0.15 in CK control soil to 8.22 ± 0.04, and 8.39 ± 0.15 in P and P-OM treatments, respectively (Table 1). The contents of SOM and NH_4_^+^-N were statistically higher in the P-OM soil than those in the CK soil (*P* < 0.05), whereas the SOM and NH_4_^+^-N content showed a decreasing trend in P soil. SOM and NH_4_^+^-N contents were elevated by 49.0% and 40.3%, respectively, in the P-OM soil compared with the CK soil. Similarly, the P-OM soil showed increase by 2.94-fold for TN. Decreased content of soil SOM, NO_3_^−^-N, NH_4_^+^-N by 31.9%, 16.9%, and 41.3% was observed in P soil compared to CK soil. The soil water content ranged from 12.62 to 24.25% with the lowest content in P soil.

**Table 1:** Soil chemical characteristics and summary of Miseq sequencing of 16S rRNA/ITS1 genes in soil samples

Samples	pH (1:2.5 H_2_O)	SOM (g kg^−1^)	TN (g kg^−1^)	Soil water content (%)	NO_3_^—^N (μg g^−1^)	NH_4_^+^-N (μg g^−1^)	Bacteria sequencing		Fungi sequencing	
							Sequencing number	OTUs number	Sequencing number	OTUs number
CK	8.43 ± 0.15a	16.48 ± 1.38b	0.35 ± 0.02c	21.12 ± 3.14a	45.25 ± 1.49a	16.71 ± 0.44b	34,983 ± 1783b	2009 ± 105b	36,789 ± 2311a	331 ± 87b
P	8.22 ± 0.04a	11.23 ± 0.25c	0.58 ± 0.09b	12.62 ± 0.88b	37.60 ± 4.65b	9.81 ± 0.41c	28,946 ± 307c	1950 ± 3b	28,450 ± 1185b	510 ± 64a
P-OM	8.39 ± 0.15a	24.56 ± 1.98a	1.38 ± 0.18a	24.25 ± 3.79a	39.75 ± 0.49ab	23.45 ± 0.34a	41,567 ± 193a	2276 ± 3a	34,373 ± 1225a	506 ± 16a

The different alphabetic letters labeled behind the data mean significant different at the *P* < 0.05 level among or between the treatments

### Richness and diversity of microbial communities

**Fig. 2:**
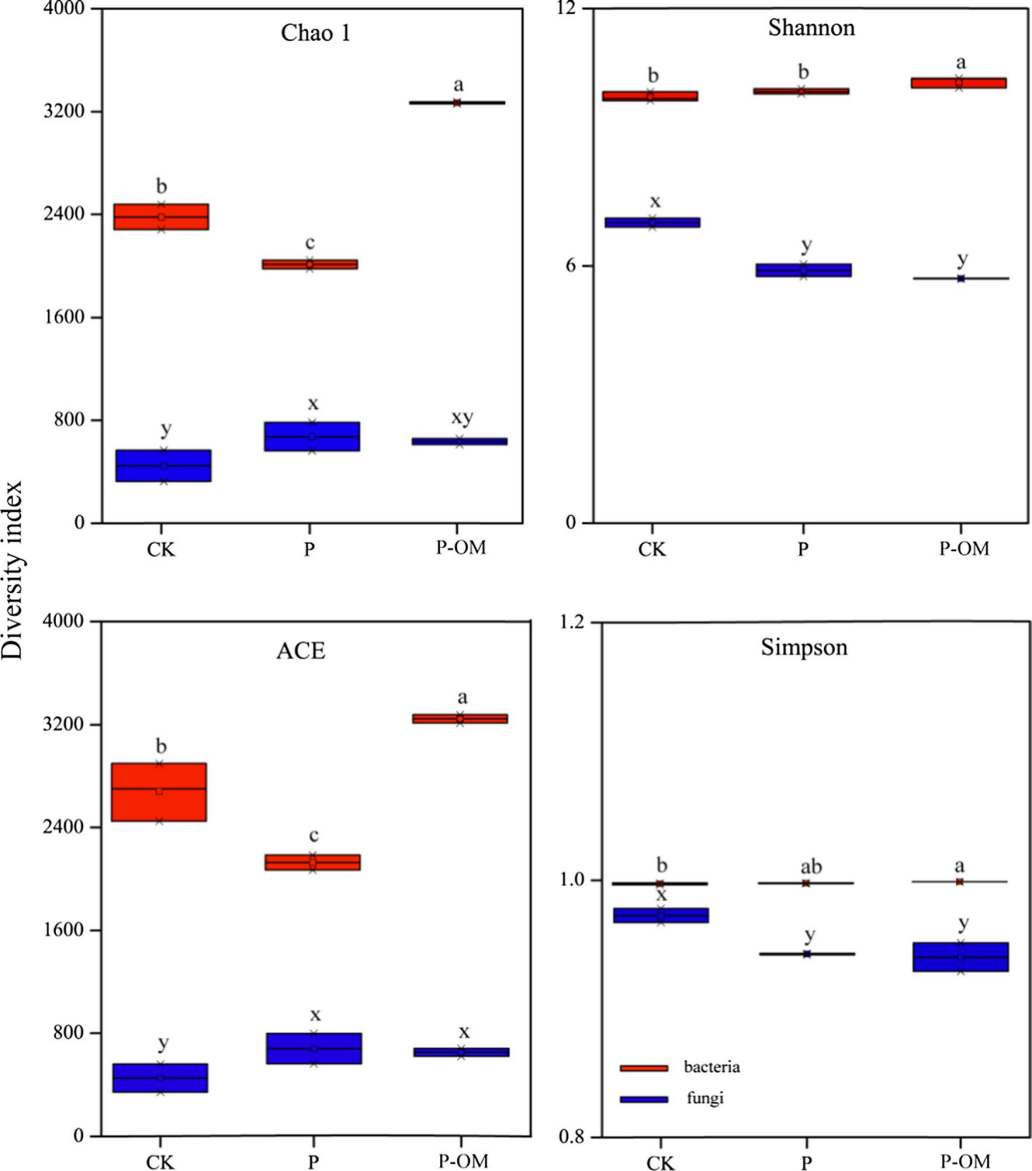
α-diversity indexes of bacterial and fungi communities. Error bars represent standard errors of the triplicate analyses. Different letters indicate significant difference soil samples (*P* < 0.05)

316,485 and 298,832 high-quality sequences for bacteria and fungi were obtained in total (Table 1). The OTUs number of bacteria showed an obvious increase from 2009 ± 105 in CK soil to 2276 ± 3 in P-OM oil, whereas a significant decrease was observed in P soil with 1950 ± 3 OTUs. The fungal OTUs number also decreased in P soil compared to CK soil. The fungal OTUs number in P-OM and CK soils showed no significant differences (*P* > 0.05). The richness Chao1 and ACE values obviously showed that the bacterial richness in P soil was higher than those in CK soil, while the opposite trend was observed for P-OM soil (Fig. 2). It appeared that the fungal Shannon and Simpson values decreased significantly in P and P-OM soils compared to CK soil (*P* < 0.05). It is noteworthy that soil TN content was found positively related with all four diversity indexes of bacteria communities (*P* < 0.05) (Table 2). A positive correlation also existed between SOM, SWC, NH_4_^+^-N and the bacterial ACE, Chao1 values. Besides, The Simpson and Shannon index of fungal communities were positively correlated with soil NO_3_^−^-N content.

**Table 2:** Pearson correlation of soil chemical properties and α-diversity indexes

Item	pH	SOM	TN	SWC	NO_3_^−^-N	NH_4_^+^-N
Bacteria						
Simpson	− 0.611	0.576	0.816**	0.480	− 0.156	0.589
Shannon	− 0.730*	0.573	0.898**	0.315	− 0.301	0.556
ACE	− 0.005	0.952**	0.708*	0.847**	0.277	0.968**
Chao1	− 0.094	0.977**	0.856**	0.770*	0.055	0.962**
Fungi						
Simpson	0.628	− 0.134	− 0.680*	0.289	0.749*	− 0.049
Shannon	0.622	− 0.253	− 0.737	0.127	0.798**	− 0.125
ACE	− 0.321	0.062	0.496	− 0.340	− 0.392	− 0.111
Chao1	− 0.304	0.033	0.462	− 0.376	− 0.394	− 0.144

*Correlation is significant at the 0.05 level

**Correlation is significant at the 0.01 level

### Bacterial community composition

**Fig. 3:**
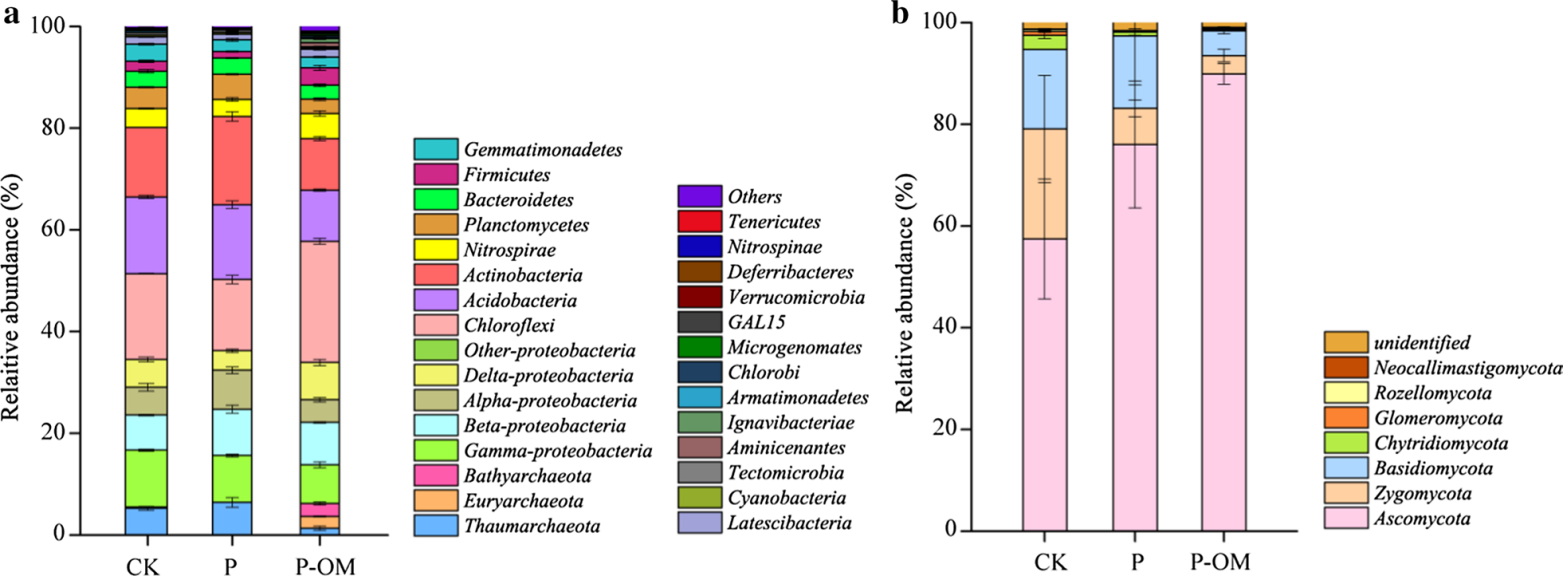
Community composition of bacteria (**a**) and fungi (**b**) in phylum level. Only *Proteobacteria* phylum was displayed in class level. The relative abundances are the average of the triplicate Miseq sequencing data

The dominant phyla across the soil samples were *Proteobacteria*, *Chloroflexi*, *Acidobacteria*, *Actinobacteria*, *Nitrospirae*, *Planctomycetes*, *Thaumarchaeota*, and *Bacteroidetes*, accounting for more than 89.42% of the bacterial sequences in the three soil samples (Fig. 3a). *Proteobacteria* represented the most abundance phylum with 29.01%, 29.84%, 27.71% of the total sequences in CK, P, and P-OM soil, respectively. The relative abundance of *Alpha*-, *Beta*-, *Gamma*-, and *Delta*-*proteobacteria* varied among different soils (Fig. 3a). *P. sinese* plantation led to 21.28%, 15.60% increase in the relative abundance of *Actinobacteria* and *Planctomycetes* in P soil compared to CK soil. The relative abundance of *Chloroflexi*, *Firmicutes*, *Nitrospirae*, and *Euryarchaeota* were significantly higher in P-OM soil than those found in CK soil (*P* < 0.05). Metastats analysis of the soil bacterial community found 9 and 25 phyla in P and P-OM soils showing significant differences compared to that of CK soil, respectively (*P* < 0.05) (Table 3).

**Table 3:** Summary of Metastats paired comparison in phylum and genus level with the significance thresholds *P* < 0.05

Sample	Level	Taxa number			Level	Sample
		CK	P	P-OM		
Bacteria						
CK	Genus	–	9	25	Phylum	CK
P	Genus	30	–	22	Phylum	P
P-OM	Genus	68	74	–	Phylum	P-OM
Fungi						
CK	Genus	–	8	9	Phylum	CK
P	Genus	111	–	6	Phylum	P
P-OM	Genus	126	92	–	Phylum	P-OM

**Fig. 4:**
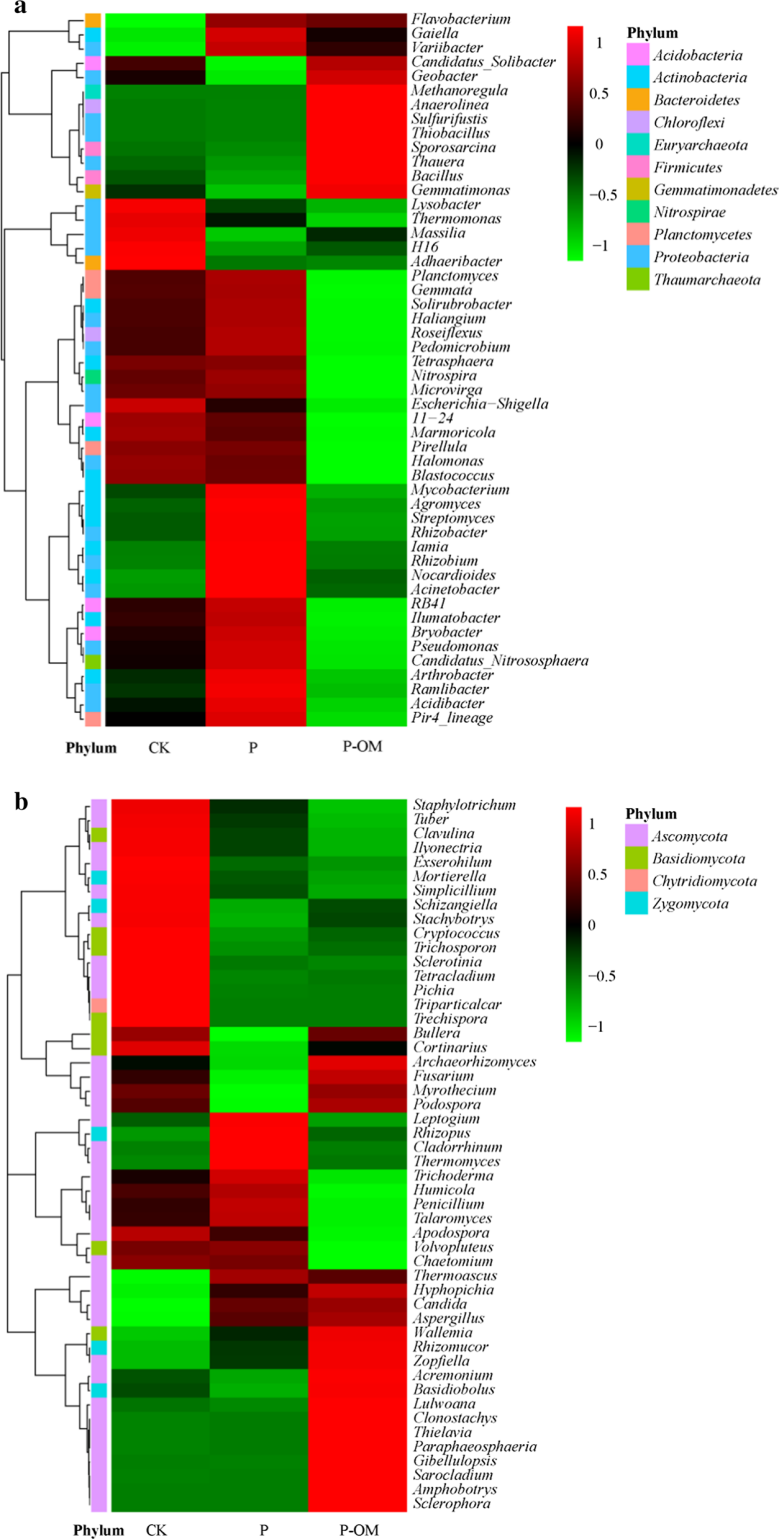
Hierarchically clustered heatmaps based on the top 50 genera of bacterial (**a**) and archaeal (**b**) composition. The relative abundance for each genus was depicted by color intensity with the legend. The genera relative abundances are the average of the triplicate Miseq sequencing data

The striking microbial community differences among treatments can be observed in the heatmap of the top 50 genera (Fig. 4a). The *P. sinese* plantation resulted in significant increased *Acinetobacter*, *Rhizobium*, *Streptomyces*, *Ramlibacter*, *Arthrobacter*, and *Mycobacterium* by 36.1-, 3.24-, 2.81-, 2.26-, 2.05-, and 1.91-fold in P soil compared to CK soil (Additional file 1: Fig. S2). Sequences belonging to *Anaerolinea*, *Geobacter*, *Thauera*, and *Bacillus* were highly enriched in the P-OM soil representing 19.4-, 9.60-, 4.10-, and 3.36-fold increase compared with the P soil, respectively.

### Fungal community composition

The predominant fungal phyla (*Ascomycota*, *Zygomycota*, *Basidiomycota*, *Chytridiomycota*) represented > 98.09% of the total sequences in the three treatments, with *Ascomycota* the most dominant phyla (57.45–89.89%) (Fig. 3b). Metastats analysis of the soil fungal community found 8 and 9 phyla in P and P-OM soils showing significant differences compared to that of CK soil, respectively (Table 3). The relative abundance of *Ascomycota* increased from 57.45 ± 11.76% in the CK soil to 76.02 ± 12.50% and 89.89 ± 2.04% in the P and P-OM soil respectively. A opposite change trend was observed in the phyla of *Zygomycota*, which were reduced by 3.05-and 5.94-fold in the P and P-OM soil. *Basidiomycota* also showed a significant decreased after *P. sinese* plantation and fertilization.

In terms of genus level, there were significant dissimilarities in the relative abundances of the dominant fungal genera in the three treatments (Fig. 4b). Specifically, the relative abundance of *Mortierella* (3.84%) in P soil was markedly lower than in CK soil (18.7%), and a further decrease in *Mortierella* relative abundance was found in P-OM soil (0.61%). The dominant genus shifted from *Mortierella*, *Volvopluteus*, and *Chaetomium* in CK soil to *Candida*, *Thermoascus*, *Hyphopichia*, *Rhizomuco*r, *Aspergillus* in P soil. *Candida* (12.3%) was the uppermost genus in P-OM soil, followed by *Acremonium* (9.38%), *Thermoascus* (5.86%). Besides, sequences affiliated with *Gibellulopsis*, *Sarocladium*, *Acremonium* increased by 119-, 53.8-, and 6.28-fold in P-OM soil.

### PICRUSt analysis of soil bacterial metabolism function

In order to compare the functional potentials of the soil samples, functional metabolisms were predicted based on the bacterial OTUs using PICRUSt (Langille et al. 2013). Genes related with nitrogen and carbon metabolic pathways were significantly more abundant in P-OM soil than in CK and P soil (Table 4). In addition, genes involved in xenobiotic biodegradation metabolism, metabolism of terpenoids and polyketides, and amino acid metabolism in P soils had the highest abundance of the three treatments (Additional file 1: Fig. S3).

**Table 4:** Relative abundance of PICRUSt-predicted reads annotated to genes for different compounds or elements metabolism

Compounds or elements metabolism	CK	P	P-OM
Nitrogen metabolism	7.05 ± 0.05b	7.08 ± 0.02b	7.18 ± 0.02a
Phosphate metabolism	2.19 ± 0.07a	2.24 ± 0.01a	2.26 ± 0.01a
Sulfur metabolism	3.27 ± 0.05a	3.16 ± 0.02b	3.18 ± 0.01b
Carbon metabolism	17.06 ± 0.11b	16.99 ± 0.13b	17.85 ± 0.11a
Metabolism of xenobiotics metabolism	1.48 ± 0.02a	1.52 ± 0.02a	1.34 ± 0.03b

Values were normalized by permillage. Means of three replicates per treatment are presented with standard deviation. The different alphabetic letters behind the data indicate the significant difference among different treatments

### Multivariate correlation analysis between bacterial and fungal communities and environmental variables

**Fig. 5:**
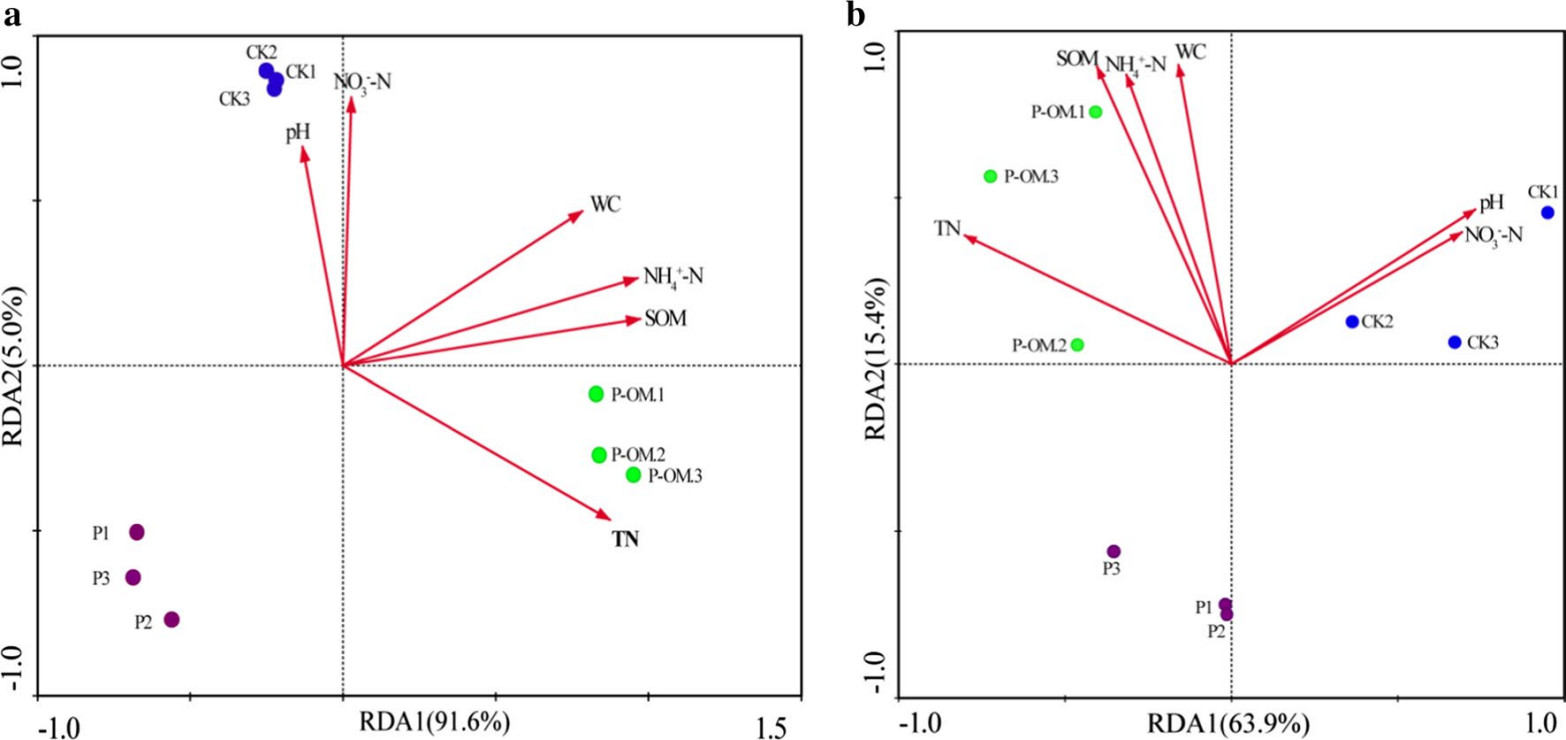
Redundancy analysis based on the bacterial (**a**) and fungal (**b**) community structure and soil chemical variables

RDA was performed based on genus-level data to determine the correlation between bacterial, fungal communities and soil physicochemical properties (Fig. 5). Figure 5a illustrated a clear separation of bacterial communities in different treatments on the first ordination axis of the plot by ANOSIM analysis (*P* < 0.01), which accounted for 91.6%. NH_4_^+^-N (*P *= 0.008), TN (*P *= 0.002), and SOM (*P* = 0.002) content were the significant determinant factors for explaining the variation in bacterial community composition (Table 5). SOM explained up to 86.0% of the total bacterial variation. For fungi, the first two canonical axes for community composition explained 63.9% and 15.4% of the variations, respectively. TN content was statistically the most significant factor determining the fungal community composition, which explained 44% of the variation. Another 35% of the variation was also significantly related with SWC and NH_4_^+^-N (*P* < 0.05).

**Table 5:** Eigen values, *F* values and *P* values obtained from the partial RDAs testing the influence of the significant parameters on bacterial and fungal communities

Parameters included in the model	Eigen value	Variation explains solely	*F* value	*P* value
Bacteria				
NH_4_^+^-N	0.02	2	3.36	0.008
NO_3_^−^-N	0.01	1	1.45	0.292
TN	0.08	8	8.37	0.002
SOM	0.86	86	44.53	0.002
Fungi				
NO_3_^−^-N	0.08	8	2.38	0.108
SWC	0.14	14	3.27	0.040
TN	0.44	44	5.54	0.004
pH	0.06	6	2.26	0.148
NH_4_^+^-N	0.21	21	3.54	0.026
SOM	0.02	2	0.83	0.532

## Discussion

The “*P. sinese*—breeding industry” ecological agriculture farm served as a model system to investigate soil microbial communities in response to 2-year *P. sinese* plantation and organic fertilization. Our results exhibited the distinct soil bacterial and fungal community patterns after *P. sinese* plantation and organic fertilization, which was accompanied by the changes of soil chemical properties.

The diversity of bacterial communities was much higher than those of fungal communities, which agreed well with the previous finding (Tian et al. 2017). The positive correlation between the bacterial diversity and soil TN content (Table 2), supported by the result from Shen et al. (2013), whereas NO_3_^−^-N content was the most dominant variable to govern the fungal diversity, which could be explained by the high susceptibility of fungi to NO_3_^−^ availability (Lilleskov et al. 2002). Here, the bacterial diversity indexes did not increase after *P. sinese* plantation. However, opposite results were reported for *Eucalyptus* plantation (Chen et al. 2013) and larch plantations (Zhang et al. 2017), indicating different plant species might have different effects on soil bacterial communities. In contrast, increased diversity indexes were observed in P-OM soil, which could be caused by the application of organic manure (Zhong et al. 2010, Zhang et al. 2012).

The bacterial communities was dominated by *Proteobacteria*, *Chloroflexi*, *Acidobacteria*, *Actinobacteria* which were also found in other soils (Stott et al. 2008; Khodadad et al. 2011). Significant increase of *Actinobacteria* and *Planctomycetes* abundance after *P. sinese* plantation was observed. *Actinobacteria* have the potential for promoting plant growth (Hamdali et al. 2008), and is known to play an important role in mineralizing N and decomposing organic material (such as chitin and cellulose) (Li et al. 2010). The pronounced increase of *Actinobacteria* after *P. sinese* plantation might be fueled by its highly developed underground roots system of *P. sinese*. The increased abundance of genera *Streptomyces*, *Arthrobacter*, and *Mycobacterium* belonging to *Actinobacteria* was also observed in the pepper plantation soil (Hahm et al. 2017). It is noteworthy that *Streptomyces* has been proposed as beneficial microbes for their capacity of plant diseases suppression (Chen et al. 2018), illustrating that *P. sinese* plantation could improve soil resistance for soil-borne diseases. Besides, 36.1-fold increase of *Acinetobacter* was highly enriched in P soil. Some species within *Acinetobacter* have been reported for their root colonization properties and broad-spectrum plant growth-promoting metabolic activities (Gulati et al. 2009). The high abundance of *Acinetobacter* in P soil might be caused by the mutualism between *P. sinese* and *Acinetobacter* (Goldstein et al. 1999).

Our results showed that the bacteria phyla *Chloroflexi*, *Firmicutes*, *Nitrospirae*, and *Euryarchaeota* significantly proliferated in P-OM soil. However, different results were reported for organic manure-involved agricultural soils where copiotrophic *Proteobacteria* dominated (Ge et al. 2008; Sun et al. 2015). The growth of anaerobic *Anaerolinea* mostly contributed the increased phylum *Chloroflexi*. This observation was consistent with previous study showing that organic fertilizer significantly stimulated the growth of *Anaerolinea* (Wu et al. 2017). It was also found that *Anaerolinea* has the metabolic potential for respiring diverse carbon compounds (Hug et al. 2013). Higher abundance of *Anaerolinea* in the P-OM soil compared to CK and P soil might be a result of high organic matter substrates (Table 1). High abundance of *Firmicutes* in P-OM soil was consistent with previous studies showing that *Firmicutes* is more abundant in soils amended with organic manures (Xun et al. 2016; Faissal et al. 2017). As previously reported (Feng et al. 2015), *Bacillus* belonging to *Firmicutes* responds most distinctly to organic manure fertilization. Yumoto et al. (2004) reported that a variety of short-chain fatty acids resulting from organic manure fermentation are excellent substrates for *Bacillus*. Other study also confirmed that the composted manure has the promoting impact on the growth of indigenous *Bacillus* in soils (Chu et al. 2007). An increased abundance of *Bacillus* in P-OM soil was also observed in this study. Moreover, members of *Bacillus* have the ability to suppress soil-borne pathogens (Weller et al. 2002) and increase soil fertility by increasing nutrient availability (Chen 2006), suggesting the potential benefits of *P. sinese*-feed organic manures application in the ecological agriculture farm. *Nitrospirae* is known to participate in nitrification (Lücker et al. 2010), which was coupled well with high NH_4_^+^ substrate and increased relative abundance of genes related with nitrogen metabolic pathway in the P-OM soil (Table 1 and Additional file 1: Fig. S4). It was found that fertilizers could enhance the abundance of carbon, nitrogen cycling genes (Nemergut et al. 2008; Su et al. 2014), which thus stimulating soil nutrient turnorver. In parallel, the content of NH_4_^+^-N and TN were statistically higher in the P-OM soil than those in the CK soil, indicating that increases in N and C availability in the P-OM soil may spur the alteration of the carbon and nitrogen-involved bacteria activities (Mickan et al. 2018).

Increased *Euryarchaeota* in P-OM soil were mostly associated with methanogens, such as *Methanoregula*, *Methanospirillum*, and *Methanosarcina* (Additional file 1: Fig. S5), which are all recognized as the key contributor for methane emission in soils (Narihiro et al. 2011; Iino et al. 2010, Gattinger et al. 2007). This observation was in line with those from other studies, showing enhanced abundance of methanogens due to organic manure application (Das and Adhya 2014; Gattinger et al. 2007). Higher abundance of genes related with methane metabolic pathways in P-OM soil compared to CK and P soil also supported this result (Additional file 1: Fig. S4). In addition, *Geobacter* and *Thauera* belonging to *Proteobacteria* were markedly enriched in P-OM soil, which was in agreement with previous studies in organic fertilized soils (Pershina et al. 2015; Luo et al. 2017). Intriguingly, *Geobacter* is famous for its crucial role in metal reduction and precipitation, and carbon cycling (Methe et al. 2003). For example, *Geobacter metallireducens* is capable of coupling the complete oxidation of organic compounds to the reduction of Fe(III), Mn(IV) and other metals (Lovley et al. 1993). According to the above results, the changes in bacterial community structure provide strong hints regarding the divergence of soil bacterial ecological function after *P. sinese* plantation and manures fertilization.

The fungal communities observed here showed dramatic variations in different soil treatments. The pronounced increased of *Ascomycota* in P (76.02%) and P-OM (89.89%) soil was observed. *Ascomycota* are particularly common in soils as the main fungal decomposer (Tian et al. 2017), and its abundance is also largely tied to plant species (Curlevski et al. 2010). *Ascomycota* was enriched after *P. sinese* plantation (Fig. 4) and *Eucalyptus* plantations (Rachid et al. 2015), whereas *Basidiomycota* rather than *Ascomycota* dominated in sub-boreal forest soil (Phillips et al. 2014). Members of *Ascomycota* are associated with litter decomposition (Ma et al. 2013) and lignocellulose degradation (Lopez et al. 2007). The increased abundance of *Ascomycota* in P soil might be caused by *P. sinese* residue which contains organic matter like lignocellulose, holocellulose, or lignin (Tian et al. 2017). Moreover, increased *Ascomycota* mainly contained the genus of *Candida*, *Thermoascus*, and *Aspergillus* which have been found to have positive effects on the decomposition of plant litter (Squina et al. 2009; Ghaly et al. 2011). For example, *Candida* showed a positive effect on the litter mass loss (Cragg and Bardgett 2001). Species within *Aspergillus* genus contains the genes for hydrolysis of xylan which is abundant in plant polysaccharide (Squina et al. 2009). Comparatively, dramatically increase of *Gibellulopsis*, *Sarocladium*, and *Acremonium* abundance in P-OM soil compared to that in P soil could be impacted by the manure fertilization, which was also observed in organic amended soils (Swer and Dkhar 2014; Gleń-Karolczyk et al. 2018). Among them, *Acremonium* can be actively involved in the decomposition of leaf litter by producing lignocellulolytic enzymes (Hao et al. 2006). Thus, the shift of fungal communities implied that the fungal groups related with decomposition could lead to the alteration of soil carbon turnover rate, which warrants further study.

NH_4_^+^-N, TN, and SOM content were the most significant factors determining the bacterial community composition in the three treatments. TN content was the most predominant factor in sharping the fungal community structure. This observation is in accordance with many other studies reporting that fungal communities are associated with changes in soil nitrogen availability (Frey et al. 2004). In the current study, the relative abundance of *Ascomycota* displayed a positive correlative with TN content (*r *= 0.92, *P* = 0.001) as reported in previous study (Weber et al. 2013). Besides, SWC and NH_4_^+^-N also contributed to the variation in fungi community composition as descried in previous studies (Weber et al. 2013; Drenovsky et al. 2004). Taken together, our data highlight the deterministic role of soil properties in shaping microbial communities.

In summary, our results demonstrate that the *P. sinese* plantation and organic manures in an ecological agriculture farm significantly altered the bacterial and fungi communities structure. Bacterial metabolism function prediction results showed the relative abundance of genes associated different metabolic pathways, such as nitrogen and carbon metabolism, varied among different soils. NH_4_^+^-N, TN, and SOM content are important factors for structuring the bacterial communities, whereas TN, NH_4_^+^-N, and SWC are steering factors to determine the fungi communities. Overall, these findings exhibit a delicate relation in agriculture practice, soil chemical properties, and microbial communities, highlighting the importance of revealing the compositional and functional diversification of microorganism in agriculture ecosystem.

## Supplementary information

**Additional file 1: Fig. S1.** (A) Overview of the ecological agriculture farm. (B) *Pennisetum sinese Roxb.* (C) Geese feed on *Pennisetum sinese Roxb.* (D) Fermentation of the livestock manures. The photos were taken by Yan He. **Fig. S2.** The abundance of bacteria (A) and fungi (B) was significantly different in different samples (P < 0.05). **Fig. S3.** The second level profile of KEGG predicted by PICRUST. **Fig. S4.** Percentage of PICRUSt-predicted reads annotated to genes for key Nitrogen, Sulfur, Hydrogen, and Methane Glutamic and Carbon from each sequencing library. RA, percent relative abundance. **Fig. S5.** Relative abundance (RA) at the genus (only for *Euryarchaeota*) level in soil samples. **Table S1.** Primers and conditions used in this study.

## Data Availability

All datasets from which the conclusions of the manuscript rely were presented in the main paper. The nucleotide sequences were deposited at the GenBank with accession numbers. The nucleotide sequences were deposited at the GenBank with Accession numbers SRR8052532–SRR8052540 and SRR8052578–SRR8052586 for the bacteria and fungi in this study, respectively.
