# Supplementary material for: High-yield grass Pennisetum sinese Roxb plantation and organic manure alter bacterial and fungal communities structure in an ecological agriculture farm
Source: AMB Express. 2020 May 6;10:86. doi: 10.1186/s13568-020-01018-2 (PMC7203373; doi:10.1186/s13568-020-01018-2)
Supplement: Supplementary file 1 — Additional file 1: Fig. S1. (A) Overview of the ecological agriculture farm. (B) Pennisetum sinese Roxb. (C) Geese feed on Pennisetum sinese Roxb. (D) Fermentation of the livestock manures. The photos were taken by Yan He. Fig. S2. The abundance of bacteria (A) and fungi (B) was significantly different in different samples (P < 0.05). Fig. S3. The second level profile of KEGG predicted by PICRUST. Fig. S4. Percentage of PICRUSt-predicted reads annotated to genes for key Nitrogen, Sulfur, Hydrogen, and Methane Glutamic and Carbon from each sequencing library. RA, percent relative abundance. Fig. S5. Relative abundance (RA) at the genus (only for Euryarchaeota) level in soil samples. Table S1. Primers and conditions used in this study. [file 13568_2020_1018_MOESM1_ESM.docx]

**Additional file**

**Fig.S1 to S4**


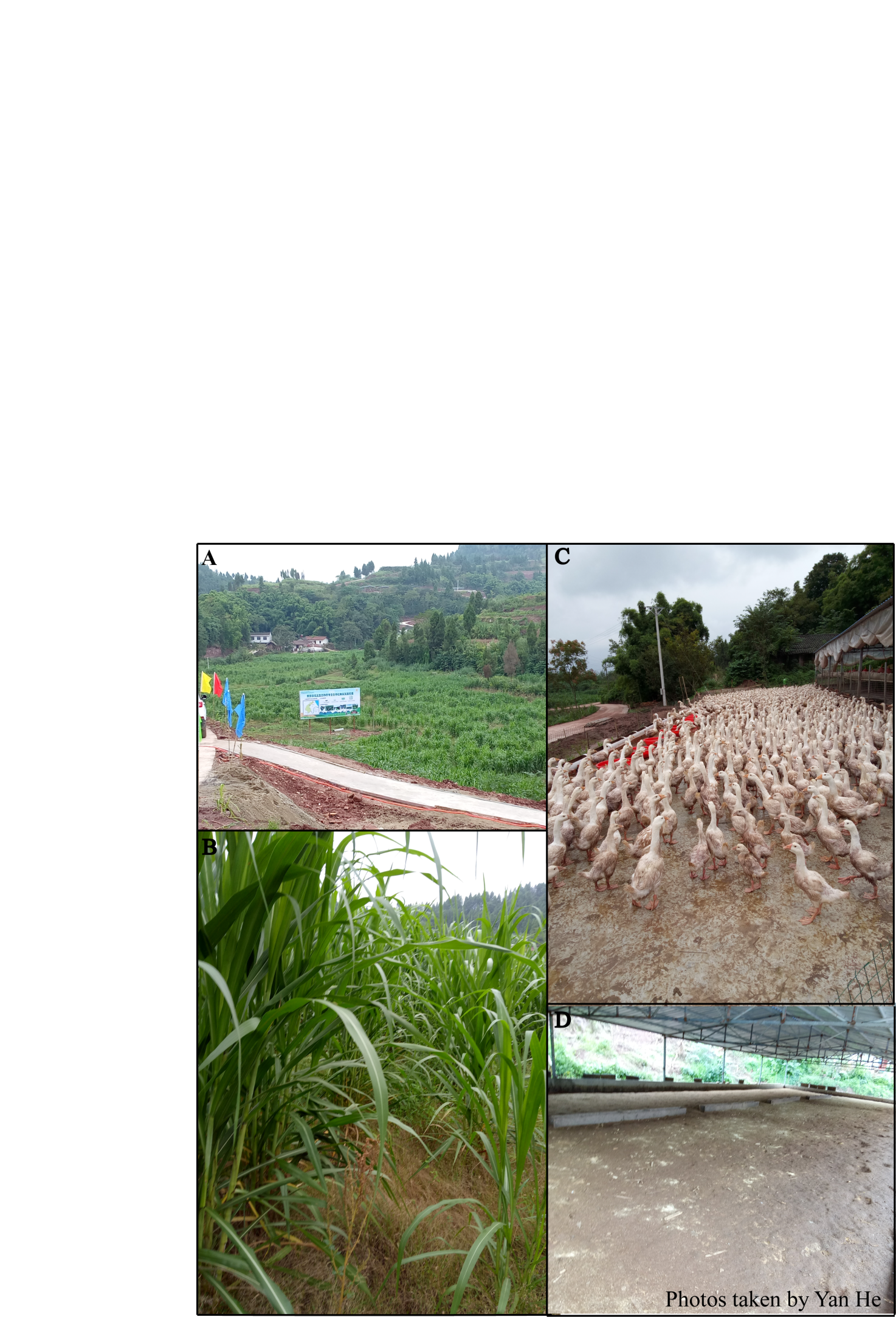


Fig. S1 (A) Overview of the ecological agriculture farm. (B) *Pennisetum sinese Roxb.* (C) Geese feed on *Pennisetum sinese Roxb.* (D) Fermentation of the livestock manures. The photos were taken by Yan He.


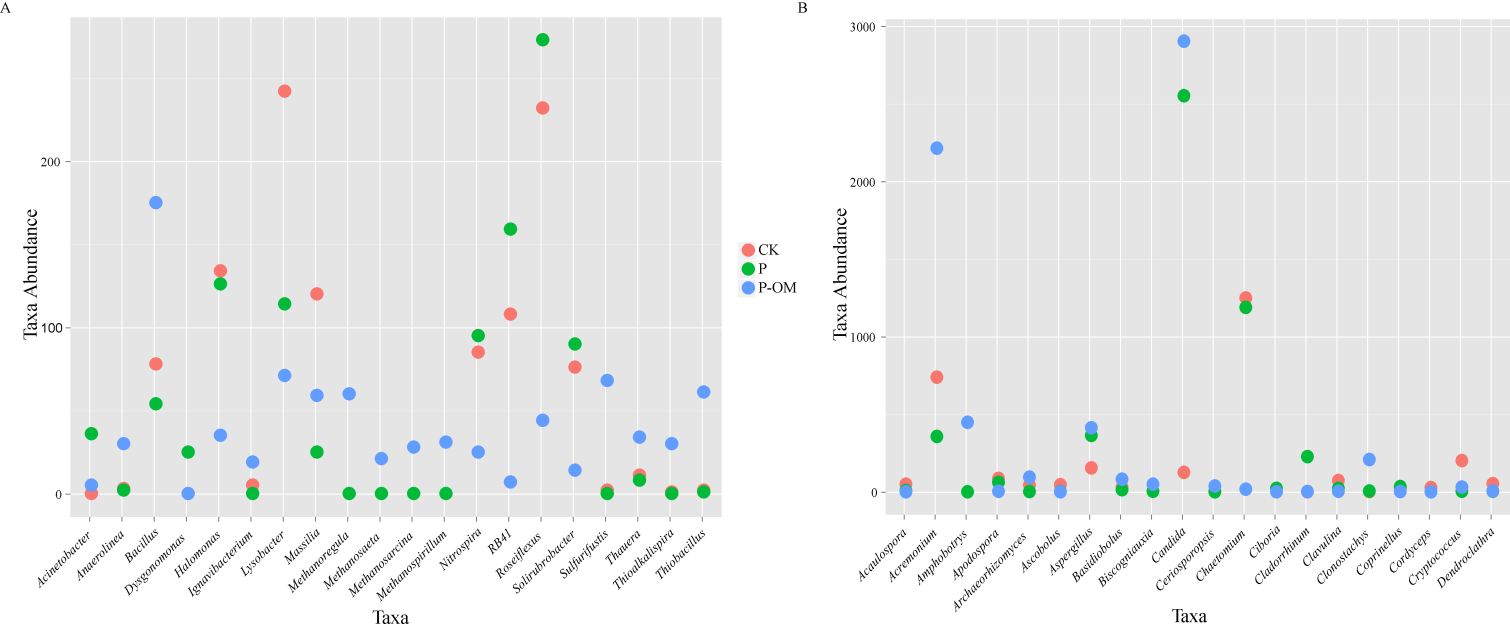


Fig. S2 The abundance of bacteria (A) and fungi (B) was significantly different in different samples (P < 0.05).


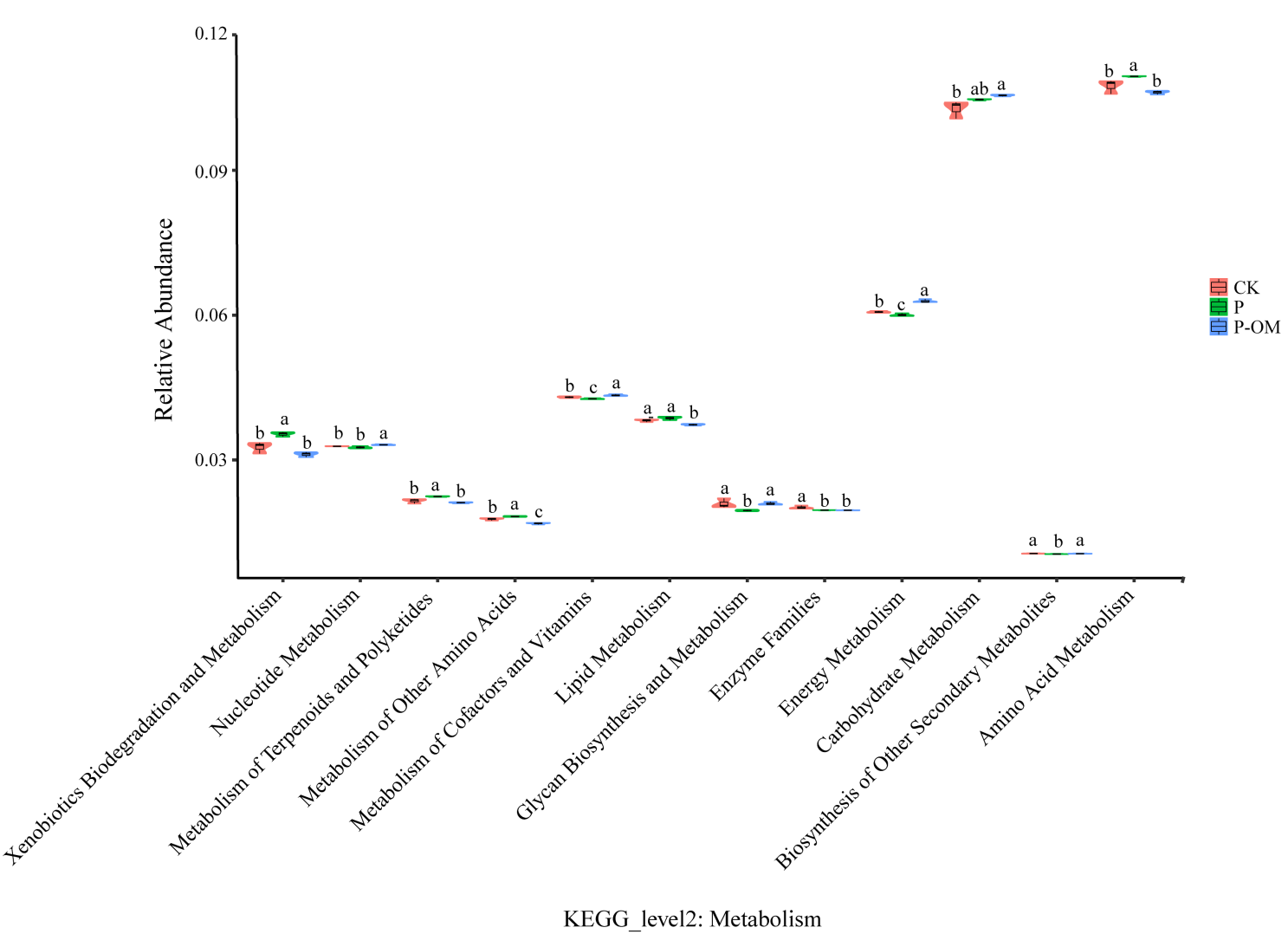


Fig. S3 The second level profile of KEGG predicted by PICRUST.


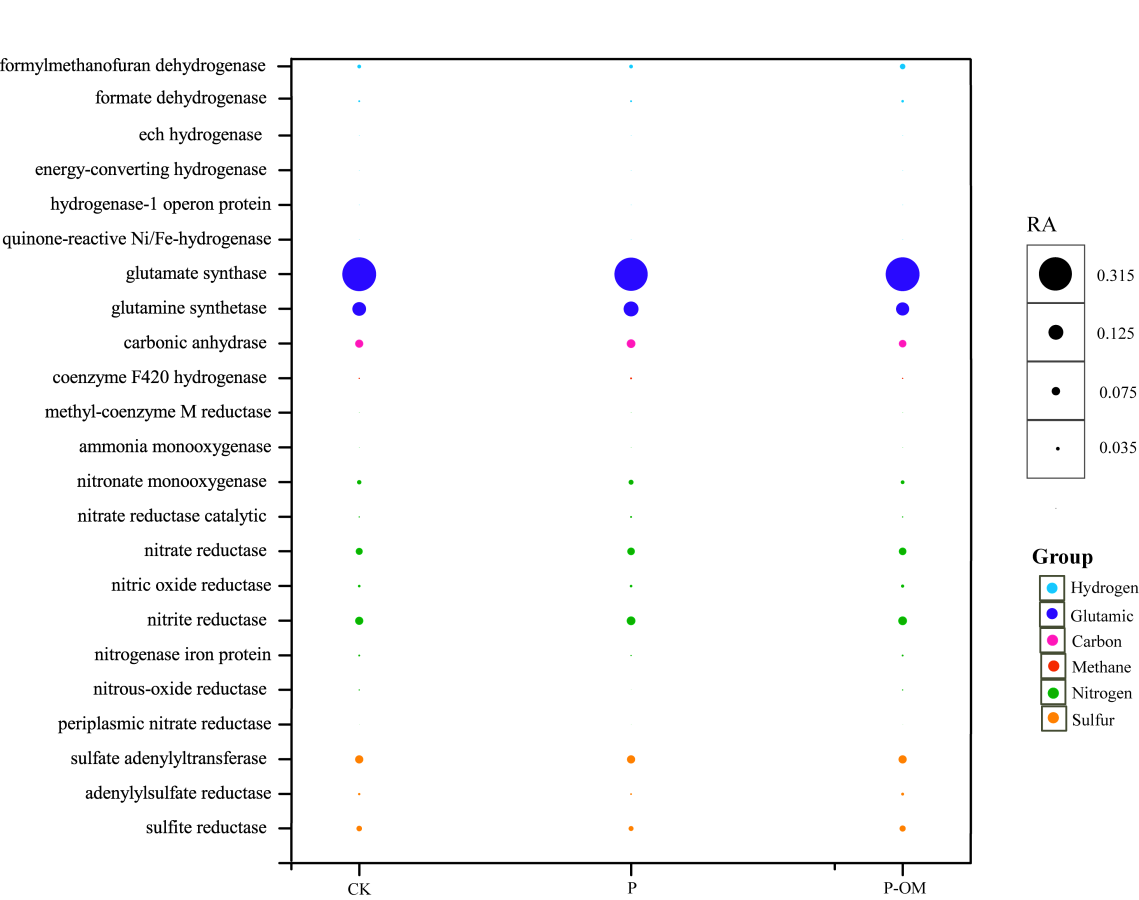


Fig. S4 Percentage of PICRUSt-predicted reads annotated to genes for key Nitrogen, Sulfur, Hydrogen, and Methane Glutamic and Carbon from each sequencing library. RA , percent relative abundance.


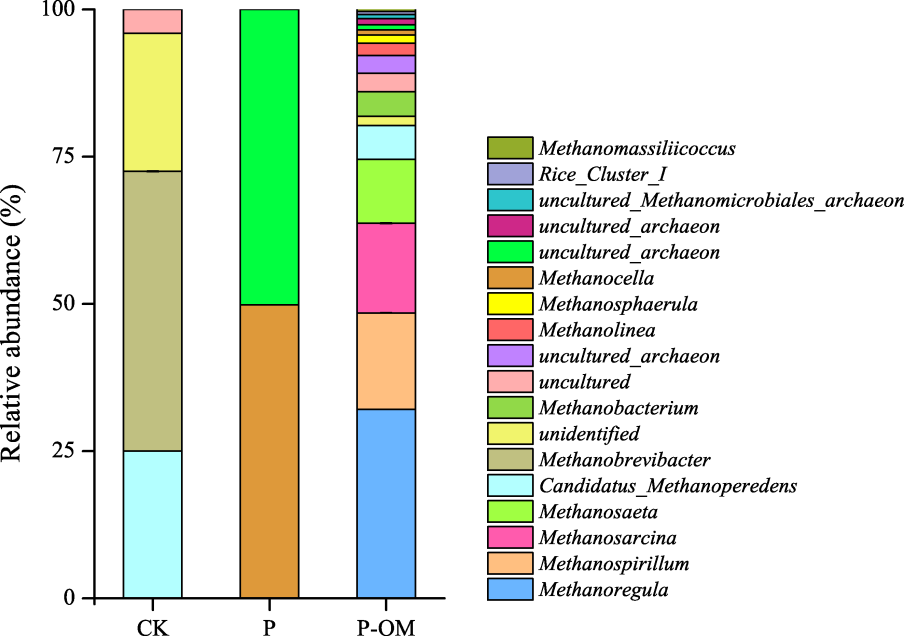


Fig. S5 Relative abundance (RA) at the genus (only for *Euryarchaeota*) level in soil samples.

**Additional Table S1**

Table S1.

Primers and conditions used in this study.

| Primer  Name | Primer sequence  （5＇-3＇） | Target  gene | Molecular analysis | Thermal  Profile | Reference |
| --- | --- | --- | --- | --- | --- |
| 515F | GTG CCA GCM GCC GCG GTA A | universal 16s  rRNA genes | Miseq  Sequencing | 98°C, 2min; 35×(98°C, 15s; 55°C, 30s; 72°C,15s); 72°C, 5min; hold at 10°C | ([Wu et al., 2017](#_ENREF_2)) |
| 907R | CCG TCA ATT CMT TTR  AGT TT |  |  |  |  |
| ITS5F | \| GGA AGT AAA AGT CGT AAC AAG G \| \| --- \| | ITS1 genes | Miseq  Sequencing | 98°C, 2min; 35×(98°C, 15s; 55°C, 30s; 72°C,15s); 72°C, 5min; hold at 10°C | ([Ding et al., 2017](#_ENREF_1)) |
| ITS1R | GCT GCG TTC TTC ATC GAT GC |  |  |  |  |

**Additional Reference**

Ding, J, Jiang, X, Guan, D, Zhao, B, Ma, M, Zhou, B, Cao, F, Yang, X, Li, L and Li, J (2017) Influence of inorganic fertilizer and organic manure application on fungal communities in a long-term field experiment of Chinese Mollisols. *Applied Soil Ecology* 111**:** 114-122

Wu, S, He, S, Zhou, W, Gu, J, Huang, J, Gao, L and Zhang, X (2017) Decomposition characteristics of three different kinds of aquatic macrophytes and their potential application as carbon resource in constructed wetland. *Environmental Pollution* 231**:** 1122
